# Certificated Nurses and Trades Unions: A Better and More Effective Alternative

**Published:** 1919-10-18

**Authors:** C. Seymour Yapp

**Affiliations:** Lake Hospital, Ashton-under-Lyne


### Certificated Nurses and Trades Unions: A Better and More Effective Alternative.

*To the Editor of* The Hospital.

Sir,—Considerable indignation has been roused by the inadequacy of the salary of £100 offered by the Birkenhead Board of Guardians for the matronship of their infirmary of 575 beds. The pre-war salary was, I believe, £80 (far too little), so to-day, based on present money value, the salary should be at least £200. The report of the "Salaries Committee" appointed by the College of Nursing to deal with salaries and conditions of employment of nurses recommends £350 as a minimum salary where the average occupied number of beds, taken over a period of not less than three years, pre-war, was above 500. We will take it that the occupied number of beds at Birkenhead was 400 (I believe it was more); the salary then recommended is £300.

Rome was not built in a day, and employers and the public have to be educated to these standards. The pecuniary value of the trained nurse, experienced administrator, practical lecturer and teacher, is valued to-day at least 50 per cent. more than in 1914. Her value was discovered to parsimonious employers not by the excellent work she did during the war, work of inestimable national value, but by the law of *demand and supply*, and unfortunately the same law is now being applied, when the supply is greater. Trained nurses will do well to reflect that underpaid matrons means underpaid subordinates—and that it is a minor evil compared to other far-reaching results. "It is scandalous," writes a nurse. "What can we do?" Nothing at present, but protest. But had we organised as we have been urged to do perpetually for the past three years, we could have dealt most effectively with these questions, with no personal trouble at all. If every trained nurse in the kingdom belonged to the College of Nursing to-day, the Birkenhead appointment would remain unfilled until such times as the salary was made adequate to the responsibility of the position. We may not have trade unionism in our profession; it would be unthinkable—but we may have professional union. A most effectual weapon is the boycott, as many members of the British Medical Association have found. That Association makes short shrift—or long—of parsimonious employers, and we can do the same, once we are entirely united. Every trained nurse who takes an underpaid appointment makes it harder for those who come after. Let no specious argument that a badly paid post is a stepping-stone to something better influence nurses. Birkenhead is no bottom rung; it is one of the top rungs of the ladder! One hundred pounds, even with the full Treasury scale of war bonus, is a totally inadequate salary for the matron of that important school. It is the more incomprehensible as the Birkenhead Board have for long been considered most progressive, especially in hospital administration. The only effective weapon we have against this very real danger of "sweating" is organisation.

The College of Nursing is a thoroughly democratic institution. Nurses can control their own economic, professional, and social advancement absolutely within three years through their College if they will, and that with perfectly legitimate weapons in keeping with the dignified traditions of our service. But I would say to all those who, like myself, are adequately remunerated and happily placed, even nurses cannot provide for old age out of noble traditions. It behoves us to help by organisation and every other means in our power those who are less fortunate than ourselves. We must ensure that those who come after shall be free from the carking anxiety which many who have helped to make our profession what it is suffered through inadequate remuneration.

Yours truly,
C. Seymour Yapp.

Lake Hospital, Ashton-under-Lyne, October 14, 1919.

